# Shortage of Nab-paclitaxel in Japan and around the World: Issues in Global Information Sharing

**DOI:** 10.31662/jmaj.2022-0179

**Published:** 2023-03-24

**Authors:** Mitsuaki Oura, Hiroaki Saito, Yoshitaka Nishikawa

**Affiliations:** 1 Department of Internal Medicine, Takeda General Hospital, Fukushima, Japan; 2 Department of Internal Medicine, Soma Central Hospital, Fukushima, Japan; 3 Department of Health Informatics, Kyoto University School of Public Health, Kyoto, Japan

**Keywords:** drug shortage, nab-paclitaxel, pancreatic neoplasms, gastric neoplasms, breast neoplasms, lung neoplasms, case study

## Abstract

There was a global shortage of nab-paclitaxel (Abraxane^Ⓡ^), a major antineoplastic agent, for a long period (from October 2021 to June 2022) because of manufacturing problems. Japan was one of the first affected countries by the depletion, and the medical institutes started to save the use of the drug in August 2021; numerous patients with gastric, breast, and lung cancer who potentially could receive benefits failed to be treated with the antineoplastic agent; thus, they opted for alternative treatments. Meanwhile, the hospitals in the United States and some countries continued to consume nab-paclitaxel at a regular pace as usual and then the worldwide depletion occurred in October 2021. Early communications about the drug shortage between authorities worldwide might have soothed the depletion; effective platforms for global information sharing would be necessary in order to secure the access to anticancer agents.

Drug shortages have been a challenge that hinders the proper delivery of healthcare and the best possible treatment to patients. It emerges when the demand overweighs the supply of the drug; it may either be due to the quick increase in demand or to the problems in supply. The depletion of paracetamol during the Coronavirus Disease 2019 (COVID-19) pandemic is an example of the former one, while the depletion of nab-paclitaxel (Abraxane^Ⓡ^, manufactured by Abraxis BioScience, LLC, Los Angeles, CA), one of the major antineoplastic drugs, is the example of the latter one, which occurred in various countries from October 2021 to June 2022. Nonetheless, it would not be possible to prioritize the delivery of drugs to those who are most in need when drug depletion occurs, unless there is global conservation and flexibility in drug use. This case study describes the chronological transition of drug depletion and related information sharing of nab-paclitaxel to help understand the issues related to drug shortage.

Nab-paclitaxel is an effective antineoplastic drug used worldwide to treat advanced breast, pancreatic, gastric, and lung cancers. The manufacturer is a subsidiary of Bristol-Myers Squibb (BMS). BMS reported to have earned $1.2 billion with the drug in 2020. (https://www.bms.com/investors/financial-reporting/product-sales-summary.html, Accessed 2021-12-23) Taiho Pharmaceutical has been importing and selling the drug exclusively to Japan since 2010.

In this case of nab-paclitaxel shortage, Taiho Pharmaceutical issued an announcement for Japanese medical professionals on August 18, 2021. It stated that “domestic supplies of Abraxane would become unstable, and the stock might run out in mid-October because of manufacturing problems.” Amid the confusion, Japan Federation of Cancer Patient Groups filed the petition to the Ministry of Health, Labour and Welfare (MHLW), Pharmaceuticals and Medical Devices Agency, and Taiho Pharmaceutical Co., Ltd. requesting detailed information disclosure and the proper distribution of the drug. Six academic societies in Japan: The Japanese Society of Medical Oncology, the Japan Society of Clinical Oncology, the Japan Pancreas Society, the Japanese Gastric Cancer Association, the Japanese Breast Cancer Society, and the Japan Lung Cancer Society issued a joint statement urging their members and hospitals nationwide to follow the instructions as follows:

1) For patients who are newly starting treatment, prioritize the usage of Abraxane^Ⓡ^ for those who have difficulty switching to alternative treatments, namely, patients who are alcohol intolerant (difficult to replace with paclitaxel) and patients with pancreatic cancer. For those with gastric, breast, and lung cancer, consider alternative treatments such as switching from Abraxane^Ⓡ^ to paclitaxel (lung cancer and gastric cancer) or switching to other treatments (breast cancer).

2) For patients who have already been treated with Abraxane^Ⓡ^, priority should be given to well-responding patients who are currently receiving it. It should be considered switching treatments for patients with gastric, breast, and lung cancer, such as changing from Abraxane^Ⓡ^ to paclitaxel.

3) Refrain from hoarding Abraxane^Ⓡ^ or alternative drugs such as paclitaxel.

MHLW made the joint statement reaching to the hospitals around Japan, which was covered by many mainstream media in Japan on August 26. These communications in the Japanese language were domestic and not aimed to reach foreign authorities.

On September 16, Taiho Pharmaceutical released a press release stating that supplies were on track until mid-November with the condition that hospitals spared the use of the drug to the patients who had alternatives. In this way, on November 11, the time limit was extended to late-January 2022; on January 24, it was extended to July. On April 19, it finally expressed that the manufacturing plant had recovered, and the supplies would return to the normal in June. There was a shortage of nab-paclitaxel for eight months in Japan, and patients with breast, lung, and gastric cancer opted to receive alternative treatments.

Drug shortage has been a worldwide problem. For example, in a survey of 820 non-federal hospitals in the United States in 2011, 99.5% of hospitals reported that they had experienced drug shortages in six months and over half of the hospitals reported >15 drug shortages ^(1)^. Moreover, it has been reported that more than half of the physicians (65.7%) in the emergency departments in Chinese hospitals have experienced drug shortages at least once a month ^(2)^. Previous interview with pharmaceutical companies in Europe states that drug shortages have been caused by a combination of factors, including manufacturing problems, shortages of raw materials, inadequate distribution and supply systems, and market trends ^(3)^.

Particularly, the shortage of anticancer drugs has been a major problem globally even before this havoc around nab-paclitaxel ^(4)^. Due to drug shortages in oncology, patients might experience interruptions and delays in treatment and replacement with alternatives, which are possibly less effective and/or with more adverse events.

Additionally, in Japan, the supply of leuprorelin acetate (Leuplin^Ⓡ^, manufactured by Takeda Pharmaceutical Co. Ltd., Osaka, Japan), a drug of choice for prostate and breast cancer, was stopped in 2020 because a plant in Japan violated Good Manufacturing Practice. This affected both all patients globally because the export of the drug halted until August 2022 ^(5)^. This infers that a problem in one particular plant may have a global effect.

Now that supply chains have been connected worldwide, pharmaceutical companies and the first countries affected by drug shortages would need to disseminate the information worldwide to warn constituents about imminent global drug shortages. Japan had faced the problem on nab-paclitaxel since mid-August 2021. To our knowledge, Australia and Canada domestically reported the shortage of the drug as well in August. There was no official statement from pharmaceutical companies, medical societies, or official bodies in the United States until the Food and Drug Administration (FDA) announced a shortage of nab-paclitaxel in October 4 (Table 1). Detailed information sharing and communication among the pharmaceutical companies, governments, and medical societies would have allowed them to reconsider the global response toward the supply disruption. Indeed, information on the drug shortages appears on the website of each country’s authorities in various formats, and integrating such information is challenging. In the absence of a platform for global information sharing, the first affected countries, pharmaceutical companies, and patient communities would be obliged to actively share the information toward worldwide. In this case, Japanese patient advocacy groups urged the pharmaceutical companies and the government to combat the drug shortage in Japan. Subsequently, a joint statement was issued by six academic societies to request medical institutions in Japan to conserve nab-paclitaxel. Likely, the COVID-19 crisis encouraged cooperation among academic societies; for example, in 2021, a joint statement was issued from four societies called “Joint Statement from Relevant Societies on Pharmacotherapy of Patients with COVID-19 and Cancer Patients under the Shortage of Oral Dexamethasone.” It would also be important to provide the information toward global communities, involve foreign academic societies, and issue a statement calling for international conservation of scarce drugs. The equitable distribution of drugs among countries would be important to avoid the depletion of essential medicines on a country-by-country basis. Still, FDA reports the shortage of >100 medicines (as of November 24, 2022.) To secure the access to anticancer treatment, timely and accurate global information sharing on drug shortage would be significant.

**Table 1.** Timeline of Events Regarding the Shortage of Nab-paclitaxel (Abraxane^®^).

Date	Event	Country	Language
Aug. 13, 2021	A website called drugshortagescanada.ca added nab-paclitaxel to the list of anticipated drug shortages, which would start from Nov. 30 because of manufacturing problems (https://www.drugshortagescanada.ca/shortage/144114, Accessed 2021-12-23).	Canada	English, French
Aug. 18	Taiho Pharmaceutical Co. Ltd. released the first report for medical professionals that anticipated shortage of nab-paclitaxel would start from October because of manufacturing problems.	Japan	Japanese
Before Aug. 19	The Australian Government Department of Health added nab-paclitaxel into its drug shortage list, and the government approved an unregistered product instead (https://apps.tga.gov.au/Prod/msi/Search/Tradename//133500, Accessed 2021-12-23).	Australia	English
Aug. 20	Japan Federation of Cancer Patient Groups filed the petition to the Ministry of Health, Labour and Welfare, Pharmaceuticals and Medical Devices Agency, and Taiho Pharmaceutical Co. Ltd., requesting detailed information disclosure and the proper distribution of the drug (http://zenganren.jp/?p=3517, Accessed 2021-12-23).	Japan	Japanese
Aug. 26	Related academic societies released a joint statement for medical professionals in Japan, requesting using other options for cancer treatment in possible cases (https://www.jbcs.gr.jp/uploads/files/info/Joint Statement.pdf, Accessed 2021-12-23).	Japan	Japanese
	Taiho Pharmaceutical Co. Ltd. released the first report for the patients and their families.	Japan	Japanese
Sep. 16	Taiho Pharmaceutical Co. Ltd. released the press release that the supply of nab-paclitaxel would meet the demand in Japan until mid-November as a result of stock adjustment and shipment adjustment.	Japan	Japanese
Oct. 4	The Food and Drug Administration added nab-paclitaxel to the drug shortage list.	United States	English
Nov. 11	Taiho Pharmaceutical Co. Ltd. released the press release that the supply of nab-paclitaxel would meet the demand in Japan until late-January under the condition that hospitals spared the use of the drug to the patients who had alternatives.	Japan	Japanese
Dec.	First communication from Bristol Myers Squibb appeared on the Food and Drug Administration website.	United States	English
Dec. 16	Taiho Pharmaceutical Co. Ltd. released the press release that the supply of nab-paclitaxel would meet the demand in Japan until late-February.	Japan	Japanese
Jan. 24, 2022	Taiho Pharmaceutical Co. Ltd. released the press release that the supply of nab-paclitaxel would meet the demand in Japan until July.	Japan	Japanese
Apr.	Bristol Myers Squibb stated that the manufacture of nab-paclitaxel returned to normal on the Food and Drug Administration website.	United States	English
Apr. 19	Taiho Pharmaceutical Co. Ltd. released the press release that the manufacture of nab-paclitaxel returned to normal and that the supplies would return to the normal in June.	Japan	Japanese

## Article Information

### Conflicts of Interest

HS received an honorarium from Taiho Pharmaceutical Co., Ltd. outside of this work. YN received personnel fee from MRT Inc. outside of the submitted work.

### Sources of Funding

This work was partly supported by JSPS KAKENHI grant number JP22K17367 (YN). The funder had no role in the preparation, review, or approval of the manuscript and decision to submit the manuscript for publication.

### Acknowledgement

This work was partly supported by JSPS KAKENHI Grant Number JP22K17367 (YN).

### Author Contributions

MO, HS and YN wrote manuscript. All author equally contributed to this manuscript.

### Approval by Institutional Review Board (IRB)

None.

## References

[1] American Hospital Association. AHA survey on drug shortages. [Internet]. American Hospital Association; 2011 Jul [cited 2022 Jul 15];[about 14 p.]. Available from: https://www.aha.org/system/files/content/11/drugshortagesurvey.pdf.

[2] Yang C, Cai W, Li Z, et al. The current status and effects of emergency drug shortages in China: perceptions of emergency department physicians. PLoS One [Internet]. 2018 Oct [cited 2022 Jul 15];13(10):e0205238. Available from: https://journals.plos.org/plosone/article?id=10.1371/journal.pone.0205238. 10.1371/journal.pone.0205238 PMC6177176 30300412

[3] Bogaert P, Bochenek T, Prokop A, et al. A qualitative approach to a better understanding of the problems underlying drug shortages, as viewed from Belgian, French and the European Union’s perspectives. PLoS One [Internet]. 2015 May [cited 2022 Jul 15];10(5):e0125691. Available from: https://journals.plos.org/plosone/article?id=10.1371/journal.pone.0125691. 10.1371/journal.pone.0125691 PMC4420462 25942432

[4] Nonzee NJ, Luu TH. Cancer policy: pharmaceutical safety. Cham (Germany): Springer; 2019. Chapter 6, The drug shortage crisis in the United States: impact on cancer pharmaceutical safety; p. 75-92. 10.1007/978-3-319-43896-2_6 30552658

[5] Wada M, Ozaki A, Miyachi T, et al. Pharmaceutical good manufacturing practice: Leuplin^®^ production in Japan identifies major international shortcomings. Invest New Drugs. 2021;39:1167-9. 33559805 10.1007/s10637-021-01080-y

